# Draft Genome Sequences of *Salmonella* Lysozyme Gene Knockout Mutants

**DOI:** 10.1128/genomeA.00519-17

**Published:** 2017-06-08

**Authors:** Narine Arabyan, Bihua C. Huang, Bart C. Weimer

**Affiliations:** a Department of Population Health and Reproduction, School of Veterinary Medicine, University of California, Davis, Davis, California, USA; b 100K Pathogen Genome Project, UC Davis, Davis, California, USA

## Abstract

Lysozyme enzymes hydrolyze the β-1,4-glycosidic bond in oligosaccharides. These enzymes are part of a broad group of glucoside hydrolases that are poorly characterized; however, they are important for growth and are being recognized as emerging virulence factors. This is the release of four lysozyme-encoding-gene-deletion mutants in *Salmonella enterica* serovar Typhimurium LT2.

## GENOME ANNOUNCEMENT

Lysozyme enzymes belong to the glucoside hydrolase 24 (GH24) family (1). GHs play an important role during infection by altering the host glycan structure to gain access to the host epithelial cells by binding to terminal monosaccharides to initiate glycan degradation (2). Lysozyme enzymes recognize host GlcNAc containing glycans in the form of N-glycans, O-glycans, glycolipids, glycoproteins, and glucosaminoglycans during infection (3) for digestion, and hence may be new virulence factors due to cleavage of the b-1,4-glycosidase bond. These GlcNAc molecules are linked to monosaccharides in the glycan via a β-1,4-glycosidic bond (4) that can be cleaved by enzymes from *Salmonella* with lysozyme activity during host association.

Lysozymes with β-1,4-glycosidase activity are also involved during the secretion of proteins, which is central for the virulence of all pathogenic bacteria (1). Gram-negative organisms translocate proteins across the peptidoglycan that is composed of linear chains of *N*-acetylglucosamine (GlcNAc) and *N*-acetylmuramic acid (MurNAc), and the alternating sugars are connected by β-1,4-glycosidic bonds (5–7). The peptidoglycan structure is a physical barrier for the assembly of macromolecular complexes and for the transport of proteins. For this reason, all bacterial lysozymes degrade the peptidoglycan to allow the assembly of type III or type IV secretion systems essential for virulence, flagella, or conjugation (8, 9). This remodeling creates gaps in the peptidoglycan necessary for the assembly of these macromolecular systems. Intracellular pathogenic bacteria, such as *Brucella abortus*, use lysozyme during the early stages of intracellular replication (8).

Four *Salmonella enterica* serovar Typhimurium LT2 lysozyme mutants (Δ*STM1028,* Δ*STM2612*, Δ*STM2715.S*, and Δ*STM3605* mutants) were constructed in the Weimer laboratory (UC Davis, Davis, CA) (2), as described by Datsenko and Wanner (10). Cultures were grown on 1.5% Luria-Bertani (LB) agar (Difco, Franklin Lakes, NJ) with 10 µg/ml chloramphenicol at 37°C and lysed (11); genomic DNA (gDNA) was extracted (12) and checked for quality (13); and sequencing libraries were constructed using the Kapa HyperPlus kit, with enzymatic-based fragmentation (13), and indexed with Weimer 384 TS-LT DNA barcodes (Integrated DNA Technologies, Inc., Coralville, IA, USA) at 192 genomes/lane. The final libraries had average sizes of 350 to 450 bp (14, 15). All genomes were sequenced on an Illumina HiSeq 4000 using PE150 (13, 16, 17) at the UC Davis DNA Technologies Core (Davis, CA). Genome sequences were *de novo* assembled using CLC Workbench version 6.5.1 (Qiagen), with default parameters.

This work was done as part of the 100K Pathogen Genome Project (http://www.100kgenomes.org), which is a large-scale sequencing consortium that uses next-generation sequencing methods to create genome databases for use in public health, food safety, and environmental science, where it is critical to capture genome diversity. This project is focused on sequencing genomes of bacteria from the environment, plants, animals, and humans worldwide, providing new insights into the genetic diversity of pathogens and the microbiome.

### Accession number(s).

All sequences are publicly available and can be found at the 100K Project bioproject (NCBI PRJNA186441) in the Sequence Read Archive (http://www.ncbi.nlm.nih.gov/sra), and genome assemblies can be found in NCBI GenBank (see accession numbers in Table 1).

**TABLE 1:** *Salmonella enterica* serovar Typhimurium LT2 deletion mutants with lysozyme activity

GenBank accession no.	SRA accession no.	Isolate name	Gene deleted	Enzyme activity	No. of contigs	Coverage (×)	Total genome size (bp)	No. of CDSs^a^
MZNN00000000	SRR5288766	BCW8410	Δ*STM1028*	Lysozyme	68	156	4,894,775	4,816
MZNO00000000	SRR5288765	BCW8422	Δ*STM2612*	Lysozyme	66	138	4,894,815	4,814
MZNP00000000	SRR5288764	BCW8423	Δ*STM2715.S*	Prophage lysozyme	67	138	4,894,604	4,807
MZYU00000000	SRR5288741	BCW8430	Δ*STM3605*	Phage endolysin	59	79	4,893,277	4,803

a CDSs, coding sequences.
